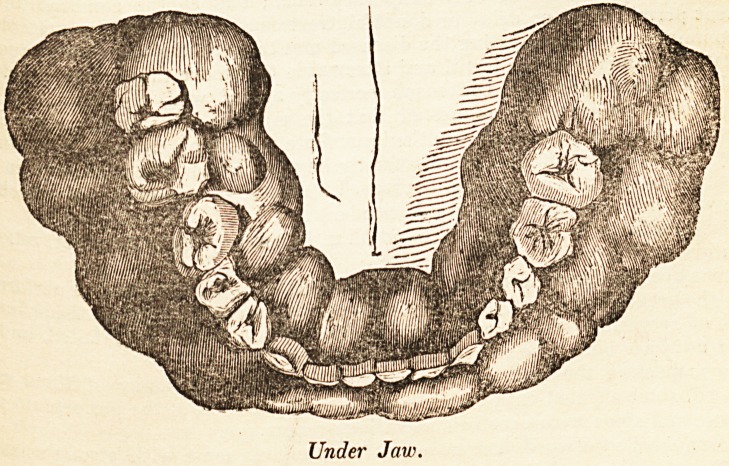# Extra-Limites

**Published:** 1843-01-01

**Authors:** 


### EXTRA-LIMITES.

Case of Extraordinary Fungous Disease of the Gums and Sockets of the Teeth, its Constitutional Effects, and successful Treatment. By *Leonard Koecker*, M.D., D.D.S , &c. &c. Surgeon-Dentist.

*Upper Jaw.*

*Under Jaw.*

During a practice of upwards of 30 years I have been more and more convinced of the various and powerful injurious influence and morbid effects which the disorders of the teeth and their adjacent parts exert over the whole animal economy, and I have, in all my writings on Dental Surgery, as well as in my practice, endeavoured to prove this fact so important to mankind; but still I have to lament that it is not yet sufficiently known, for which reason, I trust, the following case will not be considered unworthy of public attention.

Mr. Atlee, of Ealing, about 60 years of age, originally of a very robust constitution, had for nearly 30 years been a great martyr to the gout, for which he had taken various powerful medicines with only temporary benefit; he also frequently suffered from severe pain in the ears, and his hearing had become very defective.

The patient was then under the care of Mr. Dickenson of Ealing, and on consultation with Mr. Lawrence, the latter gentleman advised my being consulted.

On the 30th August, 1840, when I visited the patient, he had been bedridden for six months, and was reduced to a state of great emaciation and debility. On examining his mouth it presented a most forbidding appearance; all the teeth blackened or discolored and much furred with tartar, were imbedded in, and surrounded on all sides by an irregular fungous, and partially ulcerated mass of a deep-red, almost livid appearance, extending above half an inch broad on both sides of the teeth, and half an inch deep. The whole mouth was in a state of great inflammation, especially the diseased parts, and excessively painful even to the slightest pressure of the tongue, and his breath was extremely offensive.

The fungous excrescences in many parts extended beyond the chewing surfaces of the teeth, and hence any attempt to close them together occasioned agonizing pain, in consequence of which the sufferer was totally unable to take any solid food.

The patient was still in possession of nearly all his teeth, and with the exception of one or two of them, they were all, as far as I could ascertain, sound and firm in their sockets; but my experience having taught me that such a state of the mouth generally arises from a diseased condition of the roots of the teeth or their sockets, and other osseous structure of the jaws, I gave my opinion that the removal of the diseased mass alone would be the far more painful operation, and still be productive of only temporary relief; and as the condition of the patient permitted of no delay or doubtful treatment, I proposed, in preference, to commence by emancipating the diseased mouth from the immediate cause of irritation, namely, all the teeth, and afterwards to remove the excrescences.

Mr. Lawrence and Mr. Dickenson perfectly agreed with my views, and the patient himself earnestly requested that the most speedy remedy should be adopted, at the same time urging the immediate performance of the operation.

By the assistance of some of his family he was placed in a chair, and in the course of ten or fifteen minutes I removed 29 teeth, the extraction of which he bore with the most extraordinary fortitude.

Being replaced in his bed, he stated that he already felt somewhat relieved from his sufferings.

It may be necessary here to remark, that such an operation must be performed with the greatest care and judgment, as it is not improbable that, in the ordinary mode of removing teeth, the strength of the patient would have failed, and he could not have borne the extraction of so many.

On inspecting the teeth I found, as I anticipated, that many of them were diseased, some affected with caries, some with denudation of the periosteum and sockets, and some with exostosis in various stages.

Eleven days afterwards I removed all the fungous mass with strong scissors of different forms, and having requested to be informed of the progress of the case, and receiving repeated information that the patient was rapidly improving in health, I did not deem it necessary to see him again.

Nearly two years afterwards I visited Ealing again, and calling at the house of my patient, I was introduced to a robust, tall, healthy-looking old gentleman, whom I certainly should not have recognized as my patient. His mouth I found to be in a perfectly healthy state. He could masticate well, and articulated with so little imperfection, that his loss of teeth would not have been noticed. He had long been able to resume his public duties as parish-clerk.

He stated that since the operation he had been free from any attack of the gout requiring medical attendance; he had not suffered from the annoying pain of ear-ache, and his hearing was perfectly restored; and such was the excellent state of his health, that though he had reached the age of 62, he confidently expressed his conviction that he should "get rid" of the gout altogether.

---

Did I not fear to trespass too much upon the pages of this valuable Journal, I might add a very great number of other cases of a similar description, and not inferior in importance to the foregoing, which have been placed under my care by my various medical friends.

The following two cases seem to me, however, so very important, that I cannot refrain from relating them here in as brief a manner as possible.

### Case of Deafness cured by proper Dental Treatment.

Mr. ———, of Dublin, a gentleman holding a high office under Government, was induced, by the recommendation of Dr. James Johnson, to consult me respecting his teeth, on the 10th May, 1841.

He was about 48 years of age, and had generally, with the exception of some slight interruptions, enjoyed good health, but his power of hearing had for the last three or four years decreased to that extent that he now was almost perfectly deaf, so that I could with difficulty make myself understood by him. He had no hope indeed of ever recovering his hearing.

After examining his mouth and teeth, however, I gave it immediately as my decided opinion that the deafness had been produced by a very improper and injurious dental treatment, adopted during many years, I regret to say, by a very celebrated dentist, and I therefore held out to him the greatest hopes of an almost entire restoration of his hearing. His mouth and teeth were in a most pitiable condition, not only from dead, carious, and painful roots and teeth, which had been injudiciously left in the mouth, and had become covered with fetid tartar, attended with chronic inflammation and suppuration of the gums and sockets, but also from the irritating and baneful effects of a large set of unskilfully prepared and injudiciously attached artificial teeth.

By a perfect restoration to health of his remaining teeth, and all other parts of his mouth, and the subsequent insertion of a carefully adapted and properly inserted small set of artificial teeth, I had the satisfaction to see that my patient was perfectly restored to his hearing in three weeks, and a few days before his departure, a friend and patient of mine in the legal profession, who dined with him at a large public dinner, heard him say, when a friend of his was speaking very loudly in his ears, "Pray do not speak so loud, I hear again as well as ever, thanks to Mr. Koecker."

### Case of Lost Sight restored by proper Dental Treatmtnt.

Mrs. S———, was requested by Mr. Lawrence, in March 1840, to consult me about her teeth, giving it as his opinion that her sufferings were principally produced by the diseased condition of her mouth.

The patient was 69 years of age, and although delicate and nervous, did not particularly suffer in her general health, but for the last ten or twelve years had been gradually losing her sight, so that she had for some years required a constant guide, and had now become almost totally blind.

On a minute examination I found her mouth in a most diseased and disgusting condition. The gums were much swollen, of a red and livid appearance, and, together with the alveolar processes, in a state of much inflammation and suppuration.

Seven or eight dead roots and stumps were remaining in different parts of both the upper and under jaws, intermixed with 11 apparently healthy teeth, to which latter were fastened in different places four small sets of artificial teeth, of one to four teeth each, mounted on gold, and both constructed and inserted in the most injudicious and unskilful manner, so that some of the teeth, to which the sets were attached, had now become denuded of the gums and had been retained, probably for years, in their positions only by the gold fastenings of the artificial teeth. The artificial teeth had not been removed from their places for six or seven years, and had become, along with the natural teeth and roots, incrusted with a mass of tartar to such an extent that it was difficult to distinguish the one from the other.

Some of the roots and teeth were still retained firmly in their sockets, but the greater part were very loose.

In order to obtain a precise view of the case, it was necessary first to remove all the sets of artificial teeth, which proved to be a difficult task; I was obliged to scale off in the most careful manner the greater part of the incrusting tartar before I could accomplish this. Having succeeded however in doing so, I found it unavoidably necessary to extract every remaining root and tooth, not one of them being in such a state as to render the preservation possible.

After the restoration of perfect health to every part of the mouth, the lady was provided with a properly-constructed set of artificial teeth, which fulfilled every desired effect; and I am happy to state that she has gradually so far recovered her sight that she requires no guide, and her general health has ever since so much improved, that now, at the age of seventy, she enjoys better health than for the last ten years.

## Figures and Tables

**Figure f1:**
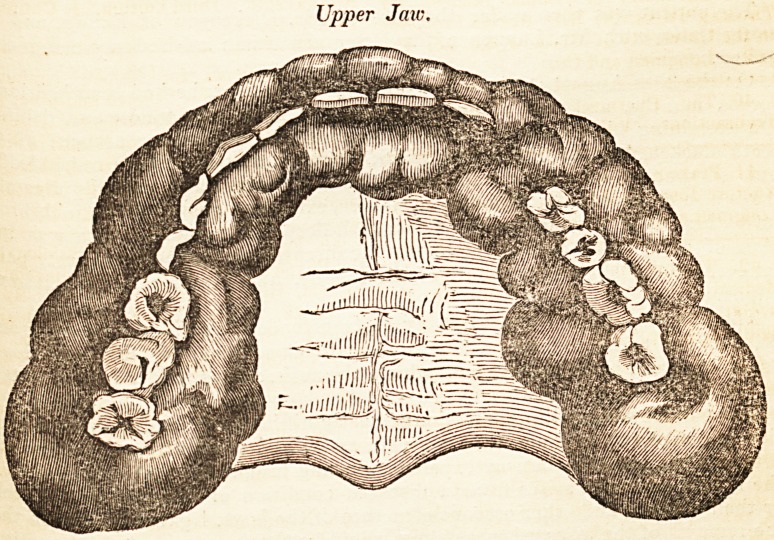


**Figure f2:**